# Bingo! Gamifying Pediatric Rheumatology Education One Square at a Time

**DOI:** 10.5334/pme.1749

**Published:** 2025-09-30

**Authors:** Miriah Gillispie-Taylor

**Affiliations:** 1 Baylor College of Medicine/Texas Children’s Hospital, US

## Abstract

**Background:**

Childhood rheumatic diseases (cRDs) are more prevalent than commonly perceived, yet medical trainees often receive inconsistent exposure to pediatric rheumatology. Many medical schools and residency programs lack affiliated pediatric rheumatologists, contributing to knowledge gaps and delays in diagnosis. With a growing workforce shortage and care disparities, innovative educational approaches are essential.

**Innovation:**

We developed an interactive, gamified bingo card to enhance pediatric rheumatology learning. This tool incentivizes engagement through structured clinical exposures, core knowledge tasks, and hands-on activities. Residents achieve “bingo” by completing five consecutive squares, each linked to key learning objectives based on American Board of Pediatrics content specifications and institutional clinical encounter data. Game mechanics—challenge, control, rules/goals, and assessment—promote self-directed learning and accountability.

**Implementation & Evaluation:**

Pediatric and medicine-pediatrics residents used the bingo card during a one-week rotation, with faculty validating completed tasks. In the 2023–2024 academic year, 50 interns received the tool, with 74% submitting completed cards. Residents checked off an average of 12 squares per week, with 31% achieving bingo. Post-rotation surveys (44% response rate) indicated the tool prioritized learning and facilitated faculty feedback. Faculty reported improved teaching efficiency by quickly identifying teaching points yet to be covered.

**Conclusion:**

Gamification enhances pediatric rheumatology education within limited clinical experiences. This tool fosters engagement, supports knowledge acquisition, and offers a scalable model for specialized graduate medical education. Future adaptations may expand game elements to further increase motivation and peer collaboration.

Childhood rheumatic diseases (cRDs) are more prevalent than many clinicians realize, with juvenile idiopathic arthritis alone affecting an estimated 300,000 children in the United States [1]. Despite this notable disease burden, exposure to pediatric rheumatology during medical training is alarmingly inconsistent. National data indicate that 33% of medical schools and 36% of pediatric residency programs lack an affiliated pediatric rheumatologist, while eight states have no pediatric rheumatologist at all [2,3]. Even in programs with a pediatric rheumatologist, optional or non-existent rotations often lead to limited or sporadic clinical experiences.

This mismatch between disease prevalence and clinical exposure directly contributes to knowledge gaps among medical trainees. Inadequate familiarity with cRDs can result in delayed diagnoses, unnecessary referrals, and suboptimal management of these conditions [4]. Consequently, the current workforce shortage in pediatric rheumatology—already projected to worsen [3]—presents an even greater challenge: without sufficient exposure to the subspecialty during training, recruitment of future pediatric rheumatologists may stall. Moreover, care disparities are amplified in regions with few or no pediatric rheumatologists, forcing families to travel long distances for consultations with orthopedics, infectious disease, or adult rheumatology, and sometimes leading general pediatricians to manage these complex diseases themselves [3].

Given the breadth of competencies graduate medical education (GME) trainees must master, innovative approaches to teaching pediatric rheumatology within an already “dense” curriculum are crucial. Attempts have been made to create open access resources for learners, and there are several pediatric rheumatology resources created with the resident trainee in mind [5,6]. However, the utilization of these tools relies on knowledge of their existence. They are generally not well advertised and often exist on password-protected websites, which makes them more onerous to access.

Game-based learning, including gamification and serious games, is an emerging educational approach that may help learners gain specialized knowledge effectively [7]. Empirical studies have shown positive outcomes with these methods [8]. Gamification is defined by Richard Landers as an application of game design attributes in non-game contexts to enhance learner engagement, motivation, and learning outcomes [9]. Leveraging this approach for pediatric rheumatology education could heighten learner interest and knowledge acquisition, especially if adopted by programs who lack formal cRD teaching. By integrating game attributes such as challenge, points, assessment and immediate feedback into traditionally didactic training modules and experiential learning, we can transform a complex subspecialty topic into a dynamic, interactive experience that engages trainees more effectively than conventional methods do [10].

Given the persistent need to enhance pediatric rheumatology knowledge among trainees, harnessing Landers’ concept of gamification offers a novel and impactful solution. By weaving game attributes into clinical education, educators can build confidence in diagnosing and managing different conditions [11]. In doing so within pediatric rheumatology, we also hope to improve trainee knowledge, which may impact patient outcomes, mitigate workforce shortages, and cultivate a new generation of physicians who are prepared to navigate the challenges of pediatric rheumatology.

Previous implementation of variations of Bingo in undergraduate (UME) and graduate medical education report success. The combination of a Bingo with multiple choice questions to create an engaging, one-time review for UME students was perceived as effective and engaging [12]. A longitudinal Bingo game covering a semester was also noted to increase engagement and impact exam scores positively [13]. These innovations utilize bingo elements but cover different topics and incorporate Bingo game differently.

## Goal of Innovation

The goal of this innovation is to create a gamified checklist, structured as an interactive bingo card, that provides pediatric residents with an organized, high-yield set of clinical exposures, core knowledge tasks, and hands-on activities to complete within the tight scheduling constraints of their rotation. By integrating game attributes (e.g., “winning” squares or completing rows) into a focused checklist, learners are incentivized to tackle real-world challenges in pediatric rheumatology—from recognizing key disease presentations to practicing essential clinical skills—in an engaging and time-efficient manner. This approach aims to increase motivation and maintain accountability for specific learning objectives while ensuring that residents acquire both foundational competencies and problem-solving abilities in a limited time span.

## Steps taken for Development and Implementation of innovation

### Context

Pediatric and medicine-pediatrics residents rotate in the pediatric rheumatology clinic four days in a week, with a fifth day reserved for continuity clinic. This schedule typically results in eight half-day clinical sessions in the entire residency training, during which each resident independently sees two to three patients per half-day. Over the course of a single week, a resident may thus encounter 16–24 patients. The relatively brief exposure and limited number of rotating residents make it challenging to provide comprehensive teaching on cRDs.

### Theoretical Framework

The innovation is grounded in Landers’ attributes of gamification, incorporating several game attributes (Table 1) into a brief clinical experience, the project seeks to increase engagement, direct learners’ focus to high-yield content, and foster self-directed exploration. Due to limited time and a small weekly cohort of residents, many other attributes (e.g., Game fiction, Human interaction, Immersion) could not be fully integrated.

**Table 1:** Gamification attributes utilized during the pediatric rheumatology experience.


ATTRIBUTE CATEGORY	DEFINITION	BINGO CARD APPLICATION

**Rules/Goals**	Clearly defined rules, goals, and information on progress toward those goals, provided to the learner.	A bingo card detailing how to “win” by completing five consecutive squares in any direction. Residents receive explicit instructions on what activity will result in completion of the square.

**Challenge**	The problems or barriers learners face, including the nature and difficulty of those problems.	Time constraints and the complexity of pediatric rheumatology cases create a built-in challenge. Residents have a single week to complete as many squares as possible to see a variety of high-yield clinical encounters to help focus learning on the variable presenting features of childhood rheumatic disease.

**Control**	The degree to which players can alter the game (or the game alters itself) and make decisions that influence outcomes.	Residents may independently select which squares to work on, guided by chart review, deciding how to prioritize patient encounters vs. knowledge-based tasks. This autonomy in choosing patient encounters and knowledge items fosters self-directed learning. Allowing some choice allows learners to prioritize things they feel are of more utility (e.g. budding pediatric pulmonologists may focus on trying to see a patient with a pulmonary renal syndrome)

**Assessment**	The method by which accomplishment and game progress are tracked.	Each square on the bingo card serves as a checkpoint. Faculty validate completion by initialing squares; the card visually tracks progress. Achieving “bingo” indicates successful engagement with high-yield content and practical application. Requiring a series of small tasks fosters more engagement than a single overarching goal such as “Learn about childhood rheumatic disease”.


Other game attributes not utilized in this intervention: action language, environment, game fiction, human interaction, and immersion. These were not included because they were not applicable to the intervention or setting. Given the constraints of the clinical exposure, these four (of nine total) attributes were able to be incorporated [13].

### Development

**Figure 1:**
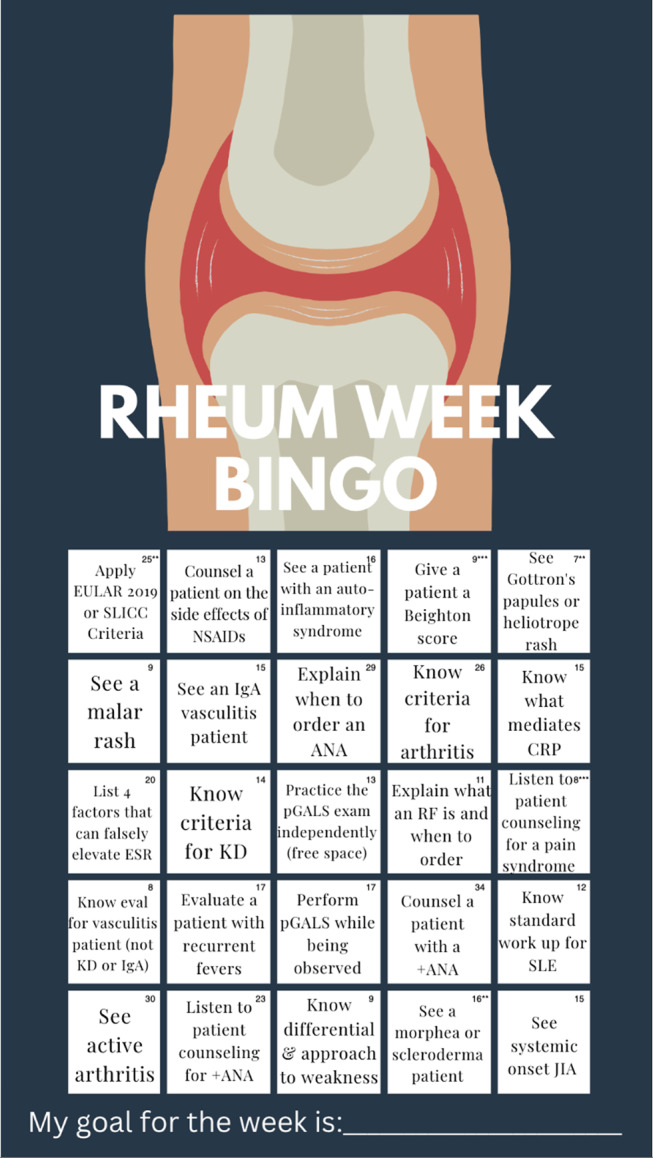
Bingo squares and frequency of utility during academic year (AY) 2023–2024. * The instructions given to trainees for squares directing them to see a particular clinical finding can be checked off if trainee sees a patient with these findings in person or if they are reviewed with a faculty member while in clinic. Squares including “apply”, “know”, “list”, “counsel”, or “explain” require verbal demonstration of the information in question. Tasks starting with “perform” should be witnessed by a faculty member. ** Initially these squares were separate, but given they are related to the same diseases processes were combined to create additional space for more high-yield topics. *** These were elements not originally included in the card. Morphea was included in the initial card, but systemic scleroderma was added in the second iteration. With the combination of SLICC and EULAR criteria and Gottron’s and heliotrope rash, respectively, “Perform Beighton score” and “Perform counseling for a pain syndrome” were added.

Topics for the “bingo card” checklist were derived from two primary sources: (1) American Board of Pediatrics (ABP) [14] content specifications for both general pediatrics and pediatric rheumatology, and (2) the institution’s electronic health record data obtained via SlicerDicer, a self-service data and exploration tool within the electronic health record, used to identify common chief complaints and diagnoses seen in pediatric rheumatology clinic. This ensured that the bingo squares would reflect both standard competencies and locally relevant clinical encounters. A list of 25 items was generated to fill the card’s 25 squares (Figure 1). Each square addressed a knowledge or clinical skill objective, and to earn “bingo,” residents must complete a row of five squares in any direction, thereby ensuring diverse content coverage (e.g., pathophysiology, diagnostic criteria, physical exam findings).

We shared the initial bingo card draft with pediatric rheumatology faculty, whose feedback prompted minor adjustments. The card’s overall design remained largely unchanged to preserve the balance between knowledge-based tasks and real-world patient encounters.

### Implementation

At the start of their weekly rotation, each resident received the bingo card in addition to a primer covering common cRDs. The instructions are to have supervisors initial the cards to serve as a stamp of completion. Residents must demonstrate knowledge or describe relevant patient encounters to a supervising faculty to mark a square as complete. For items requiring visualization of cutaneous involvement, it was acceptable to review images with a faculty member for credit. Residents were instructed to reference the bingo card throughout their clinical sessions and prioritize capturing relevant experiences. The “rules/goals” aspect of gamification was reinforced by providing clear instructions for completing squares and clarifying that “bingo” can be achieved by checking off five consecutive squares in any direction. The bingo play required faculty validation; upon satisfactory demonstration, the faculty initialled the square, ensuring accountability and reinforcing feedback on real-time learning.

In our first year (academic year 2023–2024), no prizes were given for achieving bingo. However, to further motivate residents during the current academic year, we introduced a small prize (e.g., a waterproof “I Got Bingo!” sticker). Residents had one week to see if they could meet the goal of completing a row. This duration of time and the low stakes nature of the game – there were no negative consequences for not getting bingo – did not impart undue stress on residents.

At the end of the week, residents completed a brief, anonymous survey using a five-point Likert scale, which was piloted for readability and utility on a small group of learners from the prior academic year. The survey gauges their perceptions of: enjoyment of the gamified tool, utility in prioritizing which patients to see, and perceived variety of patient encounters. This feedback informs ongoing refinements to the bingo card, currently in its fourth iteration, and to the overall educational strategy.

## Evaluation of Innovation

During the 2023–2024 academic year, 50 interns received the bingo card, and 37 (74%) returned photos of their cards. The most frequently marked squares included “Counsel a patient with +ANA,” “See active arthritis,” and “Explain when to order ANA.” Eleven residents (31%) achieved bingo by completing five consecutive squares. On average, residents checked off 12 squares per week (range: 7–20).

### Resident Feedback

Of the 50 interns, 22 (44%) completed a post-rotation survey, yielding predominantly favorable responses (Table 2). Residents reported that the bingo card guided “self-directed learning about essential rheumatologic conditions” and helped them “prioritize their learning.” One resident also noted its utility in showing attendings “what teaching points had not been covered,” thereby streamlining faculty feedback.

**Table 2:** Summary of responses from surveys collected in AY 2023–2024.


VARIABLE	N	MEAN	Std Dev	MEDIAN	LOWER QUARTILE	UPPER QUARTILE

I like using this gamified tool	22	4.23	0.97	4	4	5

I felt this tool helped me decide which patients to prioritize seeing	22	3.86	1.04	4	3	5

I felt this tool helped me see a variety of different patient types and encounters	22	4.14	0.94	4	3	5


Five-point Likert scale was included in an anonymous survey at the conclusion of the week, with overall favorable results.

### Faculty Feedback

Qualitative interviews with thematic analysis of three faculty members who frequently validated squares for trainees found that the bingo card helped them “really pay attention to what residents need to know about rheumatology as general pediatricians,” reduced redundant teaching by showing “what other people have talked about,” and provided a springboard for “asking questions to test their retention.” One faculty member observed that the volume of potential teaching points can be overwhelming, and the card helped them focus on the most critical learning objectives.

## Critical Reflection

In alignment with Landers’ attributes of gamification, we created a gamified checklist to help focus attention during a brief pediatric rheumatology experience to help trainees focus their limited time on high-yield, cRD topics most pertinent to becoming a good general pediatrician. The checklist served as a game element designed to modify behavior and cognitive processes through engagement and feedback mechanisms, ultimately supporting learning outcomes by promoting goal-directed activity within a non-game context. While this tool did allow us to capture some baseline data regarding the experiences of some trainees in our clinic which we did not have previously, participation is voluntary. Trainees reported appreciating the guidance the bingo card provided, however it sometimes required prompting from the faculty for use, and even more prompting for the card to be returned, introducing bias.

### Limited Gamification Elements

Of the nine gamification attributes identified in prior literature, only four were employed. This modest application results from the brief duration of clinic exposure, the small number of rotating residents, relatively lower volume of the pediatric rheumatology clinic compared to other subspecialties, and logistical constraints that preclude more complex design features like leaderboards or group competitions. Because only one (or very few) resident(s) may rotate during any given week, no direct peer competition can occur. This potentially diminishes some motivational aspects of gamification, although it does mitigate undue stress or competition. For rotations with at least two learners, adding human interaction would be another attribute that could be added with ease and likely create more of a sense of competition. With a larger number of learners, it would also be feasible to introduce conflicts or challenges based on clinical problems that would foster discussion. Currently our tool has many passive elements; to further increase engagement we can edit squares to require more active tasks.

### Short Rotation Span

With truly less than one week in the clinic, there is limited time for repeated interventions or iterative challenges, reducing the opportunity for more robust, longitudinal gamified experiences. In longer rotations, either 2- or 4-weeks, it would be possible to use game fiction to help with increasingly difficult concepts.

### Practical Application and Considerations

The duration of the rotation was the key driver behind the adoption of this intervention followed closely by the need to cover as much high-yield material as possible. Given that many patients who present with multiple, often vague, complaints, it is imperative to focus each clinical encounter with a tangible learning point for trainees.

## Additional File

The additional file for this article can be found as follows:

10.5334/pme.1749.s1 Supplementary File. Post-rotation surveys.
