# Supplementary material for: Bingo! Gamifying Pediatric Rheumatology Education One Square at a Time
Source: Perspect Med Educ. 2025 Sep 30;14(1):399–404. doi: 10.5334/pme.1749 (PMC12493062; doi:10.5334/pme.1749)
Supplement: Supplementary File. — Post-rotation surveys. [file pme-14-1-1749-s1.pdf]

Post-Rotation Survey

We within the Pediatric Rheumatology Division at Texas Children's Hospital are looking to conduct research on the impact of gamification of learning during our rotation. We are asking for your honest feedback to help improve learning opportunities.

This survey will investigate the usage of either a Bingo card for interns or a Passport for senior residents and medical student rotators. Willing rotators will complete one short, anonymous survey after completion of their time with the division. We will also request a copy of your Bingo card or patient encounters from the Passport. Survey responses will only be available to Dr. Miriah Gillispie-Taylor and no identifiable information will be collected.

The choice to participate is entirely yours. There is nothing to lose and no retaliation for negative feedback, if given. There is also no cost to engage in this study. Please contact Dr. Miriah Gillispie-Taylor (Miriah.Gillispie-Taylor@bcm.edu) if you have any questions about the survey. If you have additional questions about your rights as a research subject, please contact the Institutional Review Board for Human Subjects Research for Baylor College of Medicine & Affiliated Hospitals at (713) 798-6970.

Thank you,
Miriah Gillispie-Taylor, MD
Assistant Professor
Course Director, Pediatric Rheumatology
Baylor College of Medicine/Texas Children's Hospital

* Required

1. I rotated with rheumatology for ____ week(s). *

- ◯ 1
- ◯ 2
- ◯ 4

2. During my rotation, I utilized the: *

- ◯ Bingo Card
- ◯ Passport
- ◯ Neither

3. I am a: *

- ◯ Medical Student
- ◯ PGY-1 (intern)
- ◯ PGY-2, PGY-3, pr PGY-4 (senior resident)
- ◯ Other

4. I liked using this gamified tool: *

Strongly agree	Agree	Neither agree nor disagree	Disagree	Strongly disagree	N/A
◯	◯	◯	◯	◯	◯

5. I felt like this tool helped me decide which patients to prioritize seeing: *

Strongly agree	Agree	Neither agree nor disagree	Disagree	Strongly disagree	N/A
◯	◯	◯	◯	◯	◯

6. I felt like this tool helped me see a variety of different patient types and encounters: *

Strongly agree	Agree	Neither agree nor disagree	Disagree	Strongly disagree	N/A
◯	◯	◯	◯	◯	◯

7. I would recommend this tool to other uses. *

Strongly agree	Agree	Neither agree nor disagree	Disagree	Strongly disagree	N/A
◯	◯	◯	◯	◯	◯

8. Any additional feedback regarding the learning tools or rotation is appreciated and may be left here.
